# Treatment Updates for Neuromuscular Channelopathies

**DOI:** 10.1007/s11940-020-00644-2

**Published:** 2020-08-22

**Authors:** Nantaporn Jitpimolmard, Emma Matthews, Doreen Fialho

**Affiliations:** 1 Queen Square Centre for Neuromuscular Diseases, UCL Queen Square Institute of Neurology, UCL, London, UK; 2 Rehabilitation Medicine Department, Faculty of Medicine, Khon Kaen University, Khon Kaen, Thailand; 3 Atkinson-Morley Neuromuscular Centre, St George’s University Hospitals Foundation Trust, London, UK

**Keywords:** Myotonia congenita, Sodium channel myotonia, Paramyotonia congenita, Periodic paralysis, Andersen-Tawil syndrome, Channelopathy

## Abstract

**Purpose of review:**

This article aims to review the current and upcoming treatment options of primary muscle channelopathies including the non-dystrophic myotonias and periodic paralyses.

**Recent findings:**

The efficacy of mexiletine in the treatment of myotonia is now supported by two randomised placebo-controlled trials, one of which utilised a novel aggregated n-of-1 design. This has resulted in licencing of the drug via orphan drug status. There is also good evidence that mexiletine is well tolerated and safe in this patient group without the need for intensive monitoring. A range of alternative antimyotonic treatment options include lamotrigine, carbamazepine and ranolazine exist with variable evidence base. In vitro studies have shown insight into reasons for treatment failure of some medications with certain genotypes opening the era of mutation-specific therapy such as use of flecainide. In the periodic paralyses, the ability of MRI to distinguish between reversible oedema and irreversible fatty replacement makes it an increasingly useful tool to guide and assess pharmacological treatment. Unfortunately, the striking efficacy of bumetanide in hypokalaemic periodic paralysis animal models was not replicated in a recent pilot study in humans.

**Summary:**

The treatment of skeletal muscle channelopathies combines dietary and lifestyle advice together with pharmacological interventions. The rarity of these conditions remains a barrier for clinical studies but the example of the aggregated n-of-1 trial of mexiletine shows that innovative trial design can overcome these hurdles. Further research is required to test efficacy of drugs shown to have promising characteristics in preclinical experiments such as safinamide, riluzule and magnesium for myotonia or bumetanide for hypokalaemic periodic paralysis.

## Introduction

Skeletal muscle channelopathies are rare and heterogeneous genetic disorders caused by dysfunction of voltage-gated ion channels. These conditions are broadly classified as non-dystrophic myotonias (NDMs) and the periodic paralyses. Estimated prevalences range from 1 to 2.4/100000 in European cohorts [1, 2].

The NDMs are comprised of myotonia congenita (MC) due to mutations in the skeletal muscle chloride channel gene *CLCN1* encoding CLC-1 as well as paramyotonia congenita (PMC) and sodium channel myotonia (SCM) caused by mutations in the skeletal muscle sodium channel gene *SCN4A* encoding Na_v_1.4 [3]. Chloride channel myotonia can be inherited in a recessive or dominant pattern (Becker’s vs Thomsen’s myotonia) while myotonia due to sodium channel mutations is always autosomal dominant. Clinically, myotonia is characterised by delayed muscle relaxation presenting with muscle stiffness, impaired mobility and even falls. Patients also frequently report pain, fatigue and weakness [4]. Paediatric phenotypes include severe neonatal episodic laryngospasms (SNEL), which can be life threatening [5, 6]. Clinical symptoms can vary from mild to severe, with some patients only limited during certain parts of their life such as during pregnancy or when exposed to exacerbating factors like cold environment, while others have severe ongoing stiffness causing marked impairment of daily function and quality of life if untreated. Treatment therefore needs to be individualised with lifestyle changes and avoidance of triggers sufficient for some patients while many benefit from additional regular pharmacological intervention. Even in those who report being only relatively mildly affected, it can be worthwhile offering drug treatment as due to their lifelong affliction, some patients may be unaware how much myotonia is restricting their everyday life, which only becomes apparent with successful treatment. Evaluation of treatment is largely based on patient-reported improvement but the more formal myotonia behavior scale, the timed up and go (TUG) and sit-to stand time are useful indicators of treatment success even outside clinical research studies [7–9].

The periodic paralyses include hypokalaemic periodic paralysis (HypoPP), hyperkalaemic periodic paralysis (HyperPP) and Andersen-Tawil syndrome (ATS) all of which are autosomal dominantly inherited [10•]. The ion channel genes involved are the skeletal muscle calcium channel gene *CACNA1S* encoding Ca_v_1.1 (HypoPP), the skeletal muscle sodium channel gene *SCN4A* encoding Na_v_1.4 (HypoPP and HyperPP) and the inward rectifying potassium channel gene *KCNJ2* encoding Kir2.1 (ATS), which is expressed in skeletal muscles but also present in other tissues explaining the multisystem involvement in Andersen-Tawil syndrome. Although pathophysiology and triggering factors vary between these different conditions, all periodic paralyses are characterised by intermittent episodes of flaccid muscle paralysis where the majority of skeletal muscle sodium channels are in an inactivated state, unable to facilitate an action potential and therefore rendering the muscle membrane inexcitable. Most patients have normal muscle strength in between attacks in the early stages but a sizable proportion develop more persistent muscle weakness linked to a vacuolar myopathy. There remains uncertainty whether active treatment of attacks of paralysis may reduce the incidence or severity of myopathy in later life. A strong trigger of an attack in all forms of periodic paralyses is excessive or unaccustomed exercise followed by complete rest, a maneuver which is exploited in the long exercise test as described by McManis [11]. In this test, a focal attack of weakness is induced in an exercised hand muscle and this is demonstrated using neurophysiological measurements. Stress and lack of sleep are also common triggers but others are more specific to each form, e.g. fasting and intake of potassium-rich food are triggers in HyperPP and carbohydrate-rich food in HypoPP. Andersen-Tawil syndrome, the rarest form of periodic paralysis, is characterised by distinct physical features and cardiac arrhythmias in addition to attacks of muscle weakness, which are most often similar to those seen in HypoPP. A recent report from a registry of ATS analysed the cardiological aspects and treatment strategies and found that incidence of life-threatening arrhythmias was much higher than previously reported and that 24/118 patients (20%) required implantable cardiac defibrillator [12•]. This review will focus on the treatment of the skeletal muscle symptoms.

Symptoms of both myotonia and periodic paralysis typically manifest in childhood and adolescence and can therefore limit school attendance and ability to engage in all activities [13]. In addition to the long established phenotypes, we now also recognise rare forms of congenital myopathies and congenital myasthenic syndrome due to mutations in voltage-gated ion channel genes [14–16]. Indeed, there is a suggestion that mutations in the skeletal muscle sodium channel gene may be a cause of sudden infant death syndrome [17••].

**Fig. 1:**
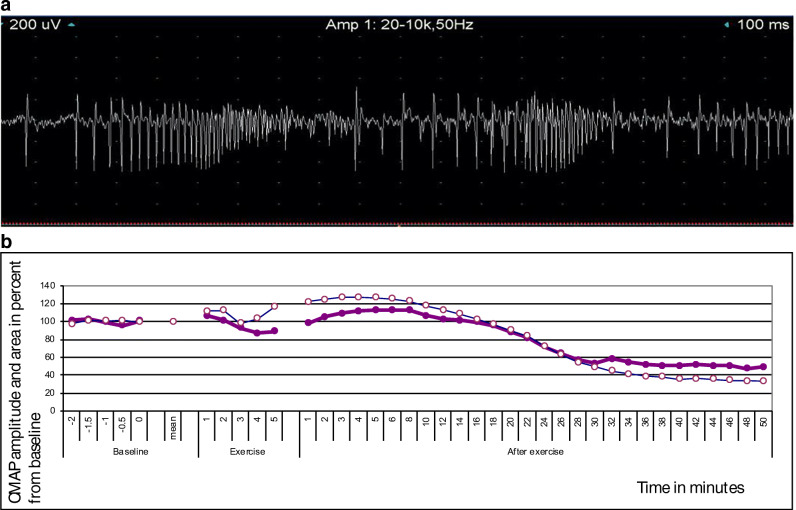
**a** EMG trace of myotonic discharges in a patient with myotonia congenita. **b** Example of the of a long exercise test (McManis) in a patient with hypokalaemic periodic paralysis. CMAP amplitudes (solid dots) and area (empty circles) are first recorded at rest, this is followed by 5 min of isometric exercise of abductor digiti minimi. After exercise, the muscle is completely at rest with ongoing CMAP recordings over 50 min. During this time, the CMAP initially increases and then shows significant gradual decrement until the end of the recording. A decrement of > 40% calculated from the maximum CMAP amplitude during or after exercise is considered abnormal; in this example, CMAP amplitude showed a decrement of 58% from peak.

The diagnosis of a muscle channelopathy is based on clinical presentation, EMG findings including exercise protocols (see Fig. 1) and genetic analysis. Imaging, muscle biopsies and provocation of major attacks are not part of the routine work-up. Nevertheless, magnetic resonance imaging (MRI) shows promise as a monitoring tool. It can differentiate between irreversible weakness associated with fatty muscle replacement versus muscle oedema which is potentially treatable and may also be a biomarker of disease progression for clinical trials [18–20].

This review will focus on the recent evidence and current treatment options of inherited skeletal muscle channelopathy with an overview of drugs, doses and level of evidence provided in Table 1.

**Table 1:** Drugs in current use for skeletal muscle channelopathies, cost estimates according to British National Formulary version 2.1.33 (2020050601) updated on 6th May 2020 level of evidence according to OCEBM Levels of Evidence Working Group. “The Oxford 2011 Levels of Evidence”. Oxford Centre for Evidence-Based Medicine. http://www.cebm.net/index.aspx?o=5653

Drug	Level of evidence	Dose	Contraindication	Side effects	Monitoring	Comments	Cost/cost-effectiveness
Myotonia							
Mexiletine	Level I	150 mg bd up to 200 mg tds, occasionally 300 mg tds	Cardiac disease	Dyspepsia, dizziness, headache, palpitations, nausea, syncope	ECG baseline and after dose change, electrolytes	Avoid in pregnancy but consider in 3rd trimester	Approx. £50,000/year for 500 mg/day
Lamotrigine	Level II	25 mg daily titrated up to 300 mg daily		Headache, skin rash, muscle pain and fatigue	n/a		Approx. £61 for 100 mg/day
Acetazolamide	Level IV	125–1000 mg/day	Acidosis, hypokalaemia, hyponatraemia	Paresthesia, fatigue, mild cognitive disturbance, nephrolithiasis	Baseline and annual renal US, electrolytes	Consider if pain prominent	Approx. £112/year for 500 mg/day
Carbamazepine	Level III	100 mg bd up to 1.2 g/day	Acute porphyria, cardiac arrhythmias, bone marrow suppression	Dizziness, ataxia, drowsiness, nausea	n/a		Approx. £53/year for 400 mg/day
Flecainide	Level III	From100 mg per day, titrate to 100 mg bd	Cardiac disease	Blurred vision, worsen congestive heart failure, arrhythmias	Frequent ECG monitoring	First line for SNEL, consider in Mexiletine unresponsive SCM	Approx £115/year for 200 mg/day
Ranolazine	Level III	500 mg bd to 1000 mg bd	Heart failure, prolonged QT interval	Constipation, headache, dizziness	ECG baseline		Approx £1300/year for 2000 mg/day
Periodic paralysis							
Acetazolamide	Level III	125–1000 mg/day	Acidosis, hypokalaemia, hyponatraemia	Paresthesia, fatigue, mild cognitive disturbance, nephrolithiasis	Baseline and annual renal US, electrolytes		Approx. £112/year for 500 mg/day
Dichlorphenamide	Level I	50–200 mg/day	Acidosis, hypokalaemia, hyponatraemia	Paraesthesia, fatigue, mild cognitive disturbance and nephrolithiasis	Baseline and annual renal US, electrolytes		*
K^+^ sparing diuretic (for HyoPP)	Level IV	Spironolactone 25–100 mg/day Amiloride 5–20 mg/day Triamterene 50–150 mg/day	Addison’s disease, hyperkalaemia	Gastrointestinal, dry mouth, dizziness, hyperkalaemia, specifically with spironolactone - gynaecomastia, menstrual disturbance and erectile dysfunction	Electrolytes	No evidence of superiority for single drug, choice dictated by availability and side effect profile	Spironolactone 25 mg/day approx. £16/year, Amiloride 5 mg/day approx. £442/year, Triamterene 50 mg/day approx.. £510/year
Thiazide diuretics (for HyperPP)	Level IV	Hydrochlorothiazides 25 mg–100 mg/day Bendroflumethiazide 2.5–10 mg/day	Addison’s disease, hypercalcaemia Hypokalaemia, hyponatraemia, symptomatic hyperuricaemia	Gastrointestinal, dizziness, dry mouth	Electrolytes		Bendroflumethiazide 2.5 mg/day approx.. £8/year
Potassium supplement	Level IV	1 mEq/kg up to 200 mEq/day (acute attack) 30–60 mEq/day, not exceeding 100 mEq/day (prophylaxis)	Hyperkalaemia, heart block, gastric ulcers	Hyperkalaemia, gastrointestinal	Electrolytes	Sustained release preparation for prophylaxis but to avoid in acute attack	**

*Dichlorphenamide not listed in British National Formulary, high cost

**Oral potassium supplements not listed in BNF, low cost

## Treatment of myotonia

### Pathophysiology

Physiologically myotonia can be interpreted as an increase in muscle membrane excitability. On a channel level, this is due to gain of function with respect to sodium channel mutations or loss of function in the case of chloride channel mutations. Pharmacological treatment therefore theoretically could aim at increasing chloride conductance and/or reducing sodium channel openings. In practice, there have been no successful chloride channel openers and the majority of pharmacological agents used to treat myotonia are sodium channel blockers regardless of whether this is the primary underlying abnormal channel.

Voltage-gated skeletal muscle sodium channels open rapidly in response to the neuromuscular endplate depolarisation reaching a certain threshold. This mediates an immediate sodium influx reflected in the fast upstroke of the action potential. Sodium channels then become rapidly inactivated (fast inactivation) and are unavailable for further opening until membrane repolarisation has occurred and the channel returns to a resting conformation. In skeletal muscle, repolarisation occurs via voltage-gated potassium channels opening in response to membrane depolarisation. Skeletal muscle cells are very large structures and require the T-tubule system (membrane invaginations) in order to allow the action potential and excitation-contraction coupling to occur simultaneously throughout the whole cell. While T-tubules are connected to the extracellular space, they represent a diffusion barrier and potassium exiting the cell during repolarisation accumulates and becomes a depolarising factor as the reversal potential for potassium is shifted. This is counterbalanced by chloride channels. In the context of reduced chloride conductance, the after-depolarisation generated by the potassium accumulation is sufficient to elicit bursts of spontaneous action potentials which present as myotonia. The after-depolarisation also enhances a different type of sodium channel inactivation (slow inactivation) which may be the reason why myotonic discharges discontinue after a period of time following contraction and may explain the phenomenon of warm-up, a lessening of muscle stiffness experienced by patients with repeated muscle activation, most commonly seen in myotonia congenita. Enhancement of both fast and slow inactivation of sodium channels is the most prominent drug treatment targets at present.

#### Diet and lifestyle

##### Non-dystrophic myotonia

Exercise or rest after exercise can be a major trigger of myotonia but some activity is essential for a healthy lifestyle. It is important therefore to find a balance between activity that can be accomplished without provoking symptoms and inactivity, which can not only exacerbate myotonia but also lead to an increased risk of other co-morbidities. Reassuringly, endurance training improved fitness and maximal workload performance in six patients with myotonia congenita but did not change myotonic symptoms in a clinical trial of stationary bike training for up to 30 min, 3 times a week for 10 weeks [21]. This is in line with growing evidence of safety of exercise in a range of neuromuscular conditions [22]. Patients with myotonia should avoid sudden forceful contraction, i.e. sudden movement and instead gradually increase activity to promote warm-up. Patients who experience paradoxical myotonia (PMC, HyperPP and some with SCM) may require rest periods during certain activities but they often also experience profound cold exacerbation which can be mitigated by choosing appropriate clothing and preference for indoor activities during colder seasons. This advice is probably equally applicable to most patients with myotonia as cold was also reported to exacerbate symptoms in over 60% of those with myotonia congenita in a prospective study [23]. A particular risk is swimming in cool water where an increase in stiffness could be life threatening.

Alteration in diet does not affect the majority of patients with myotonia but some patients with underlying sodium channel mutations including those also suffering from HyperPP may be sensitive to a potassium-rich diet [23].

#### Pharmacologic treatment

##### Pharmocological treatment of myotonia

1. Mexiletine
  Mexiletine is considered the first-line drug in the treatment of non-dystrophic myotonic disorders [24] and has relatively recently been licenced in Europe for this indication with orphan drug designation. While this has resulted in a more reliable supply, it has also increased the cost significantly which may be prohibitive for some prescribers. Mexiletine is a class Ib antiarrhythmic voltage-gated sodium channel blocker, that was initially developed as a treatment for ventricular arrhythmias with subsequent use in long QT syndrome. It is favoured over other class Ib drugs such as lidocaine and tocainamide due to its better side effect profile. Its main action is through enhancement of the fast inactivation of sodium channels resulting in use-dependent block. Mexiletine interacts with a number of sodium channel isoforms and has similar affinities to Na_v_1.4 in skeletal muscle and Na_v_1.5 in cardiac muscle explaining the antimyotonic and antiarrhythmic properties [25]. Mutations specifically around the pore region may affect binding of mexiletine and this could explain some of the lack of effect in patients with certain types of sodium channel mutations (HyperPP, PMC, SCM) [25–29].
  A randomised, double-blind, placebo-controlled, cross-over, multi-centre study of 59 patients with NDM showed that mexiletine 200 mg three times a day significantly reduced patient-reported stiffness and handgrip myotonia on clinical exam and increased quality of life scores [30]. Gastrointestinal complaints were the most common side effects affecting 38% of the subjects. Two participants experienced transient bradycardia but these did not require stopping the treatment. Randomised controlled trials can be a difficult undertaking in rare conditions due to the patient numbers required but an elegant aggregated N-of-1 trials study design also supported the effectiveness of mexiletine [31••]. This trial design may be beneficial for evaluating interventions in other chronic rare diseases. It is possible that mexiletine may improve transient weakness in recessive myotonia congenita as well [32], but this was not specifically assessed in the above trials. An earlier single-blind placebo-controlled trial comparing mexiletine with three other antimyotonic drugs in a mixed group of patients with dystrophic and non-dystrophic myotonia also showed mexiletine to be effective in symptom control [33].
  A retrospective cohort study examined side effects of long-term mexiletine use (mean follow-up of 4.8 years) in skeletal muscle channelopathies [34]. One or more adverse events were reported by around 52% of patients. Dyspepsia was the most common symptom but adjunctive treatment with proton pump inhibitors ameliorated this in most. Other adverse events were headache, palpitations, nausea and syncope but there were no serious adverse events and all side effects resolved when medication was discontinued [34, 35]. ECG parameters between baseline and highest mexiletine dose levels did not change significantly and overall frequent ECG monitoring when on a stable dose of mexiletine was not deemed necessary. Instead, it was recommended that an ECG may be performed if not done within the past 2 years or with every dose change.
2. Lamotrigine
  Lamotrigine, an anticonvulsant drug, is a voltage-gated sodium channel blocker. It can reduce muscle membrane hyperexcitability by slow binding to the fast-inactivated state of voltage-gated sodium channels and shifting of the voltage-gated dependence of inactivation to more negative potentials [36–38]. Lamotrigine has been investigated in a double-blind, cross-over randomised placebo-controlled trial of 26 patients with genetically confirmed myotonia congenita and paramyotonia congenita [39•]. Oral lamotrigine was given once daily at increasing doses from 25 to 300 mg over 8 weeks. The self-assessed myotonia severity according to the Myotonia Behavior Scale (MBS) reduced by 29% with the number needed to treat 2.6. The effect size was unrelated to genetic diagnosis. Lamotrigine also improved clinical myotonia and quality of life in SF-36 domain of a physical function. The most common side effects were headache, skin rash, muscle pain and fatigue, which were considered acceptable and reversible after stopping the medication. Lamotrigine presents a suitable alternative if mexiletine is not effective (as it acts in a different manner), or if its use is prohibited by side effects or cost.
3. Acetazolamide
  Acetazolamide is a carbonic anhydrase inhibitor and its main use in skeletal muscle channelopathies is for the prophylactic treatment of periodic paralysis. It does, however, also have some antimyotonic properties demonstrated in a small open-label study of nine patients with NDM [40] as well as other case reports of chloride and sodium channel myotonias [4, 41, 42]. Indeed, one form of sodium channel myotonia has been labelled acetazolamide-responsive myotonia because of the effectiveness of the drug [43]. It has been suggested that the drug has a direct effect on skeletal muscle chloride channels resulting in an increase in chloride conductance due to intracellular acidification [44]. It can be a useful choice in patients with overlapping symptoms of periodic paralysis and myotonia. In our experience, acetazolamide can also be helpful if there is prominent pain associated with myotonia especially if used in combination with mexiletine. A pre-treatment renal ultrasound scan and yearly ultrasound monitoring are strongly recommended due to the increased risk of nephrolithiasis especially in those over the age of 40 [45•].
4. Carbamazepine
  Carbamazepine has been in use for decades as an anticonvulsant and is an archetypal voltage-gated sodium channel blocker. There are case reports with both sodium and chloride channel–related NDM showing an improvement with carbamazepine [46–49]. Many of these are from the paediatric field, possibly a reflection that paediatric neurologists are more comfortable with using carbamazepine, which is a common anticonvulsant, at a younger age compared with other sodium channel blockers. A small double-blind controlled trial of carbamazepine versus diphenylhydantoin in six patients with myotonic dystrophy also demonstrated the antimyotonic effect of carbamazepine [50].
5. Flecainide
  Flecainide is a class Ic antiarrhythmic drug. In vitro evidence points towards specific block of human skeletal muscle sodium channels during repetitive depolarization with higher potency compared with mexiletine [51, 52]. Although quite effective, there is some hesitation in using this drug due to the well-known pro-arrhythmic risk in patients with structural heart problems based on the Cardiac Arrhythmia Suppression Trial study result [53]. The incidence for cardiac disease increases with age and as treatment of myotonia is often lifelong, this risk increases over time which probably explains why there are several case reports detailing the benefits but in practice, number of patients with NDM on long-term flecainide treatment is low. The clinical evidence for flecainide is based on case reports mostly in patients with sodium channel mutations including long-term follow-up data of a family of eight patients with PMC [54–56]. A recent pharmacogenetic study demonstrated that *SCN4A* mutations clustered near the fast inactivation gate of Na_v_1.4 resulted in reduced inhibition by mexiletine (which stabilises the fast-inactivated state) but mutant channels remained sensitive to flecainide, which binds preferentially to open sodium channels and its effect is less dependent on fast inactivation gating [57•]. Examples include the common PMC mutation p.T1313M and the SCM mutation p.G1306E often associated with a severe myotonic phenotype (myotonia permanens). The latter mutation is also a common cause of severe neonatal episodic laryngospasm (SNEL) for which flecainide can be regarded as the first-line treatment [6, 56, 58, 59]. Nevertheless, the propensity for triggering life-threatening arrhythmias was illustrated by a recent case report of a patient with SCM due to p.G1306E and Brugada syndrome [60] and careful ECG monitoring is essential.
6. Ranolazine
  Ranolazine, an anti-anginal drug, demonstrated antimyotonic properties in both myotonic congenita and paramyotonia congenita model [60–62]. In contrast to mexiletine and lamotrigine, which enhance fast inactivation of voltage-gated sodium channels, ranolazine enhances slow inactivation and blocks persistent voltage-dependent sodium inward current [61–65].
  An open-label pilot study of thirteen patients with MC treated with ranolazine resulted in significantly improved self-reported severity of stiffness and reduced myotonia duration on electromyography. Measurements were compared between baseline and four weeks. The initial dose was 500 mg twice a day which was titrated to a maximum dose of 1000 mg twice a day. [66•] The same group demonstrated efficacy in a similarly designed open-label trial of ten patients with paramyotonia [67•] with additional significant effect on weakness and pain. Side effects in both trials were mild (constipation and light headedness) and led to dose restriction in one patient in each trial.
7. Miscellaneous
  There are a number of other agents with reports of antimyotonic properties mostly demonstrated in small studies or case reports of myotonic dystrophy patients, including antiarrhythmics tocainamide and procainamide; anticonvulsants such as phenytoin, antidepressant medications imipramine, clomipramine and amitriptyline; and other agents including taurine, quinine, dantrolene and dehydroepiandrosterone sulfate [for review, see 68]. Most of these are now obsolete based on alternatives with better effect and side effect profile and some have been withdrawn altogether.
8. Trends
  While there are a number of treatment options for myotonia, not every patient achieves optimal symptom control due to lack of efficacy, side effects and contra-indications precluding the use of some drugs or simple lack of access or high cost. The need and search for additional antimyotonic compounds therefore continues.
  Safinamide is a known neuronal sodium channel blocker which has been used as an add-on therapy for Parkinson’s disease. A recent report strongly suggests it has potential as an antimyotonic disorder based on its performance both in in vitro cell models as well as in a rat model [69•]. The mechanism appears to be similar to mexiletine with possibly higher potency and the effect in the in vivo rat model occurred at a plasma level of concentration which has been shown to be safe and tolerable in humans. Further studies will be required to investigate this potential alternative myotonia treatment.
  Rilulzole and lubeluzole, two benzothiazolamines with known voltage-gated sodium channel blocking effects, have also shown promising antimyotonic properties in a rat myotonia model [52]. There is concern about QT prolongation with lubeluzole but riluzole has a good safety profile as a licenced drug used in patients with motor neuron disease. Its neuroprotective activity is thought to be mediated via inhibition of persistent sodium current, a mechanism which may also reduce muscle hyperexcitability [70].
  Experimental studies have shown that reduced concentrations of extracellular Mg^2+^ and Ca^2+^ ions exacerbate myotonia due to toxin-induced ClC-1 chloride channel inhibition in isolated human [71] and rat [72] skeletal muscle fibres. Even small concentration shifts within the range of normal had an effect on myotonia severity thought to be due to a depolarizing shift in the Na_v_1.4 activation [72]. A single case report showed Mg^2+^ supplementation helped reduce weakness and myotonia in a patient with an autosomal dominant sodium channelopathy due to the SCN4A p.I693T mutation with clinical phenotype overlap between paramyotonia congenita and hyperkalaemic periodic paralysis [73]. This 19-year-old male patient also had childhood-onset aplastic anaemia treated by multiple courses of immunosuppressive therapy and then an allogeneic hematopoietic stem cell transplant. Post-transplantation symptoms of episodic muscle weakness and myotonia were aggravated in the setting of hypomagnesemia and hypocalcemia. Replacement of Mg^2+^ to the normal reference range not only restored the serum magnesium but also the serum calcium level and helped the patient remain symptom-free more than 18 months.

#### Pregnancy and anaesthetic considerations

Pregnancy and menstruation can be regarded as aggravating factors of both myotonia and periodic paralysis [23, 74]. Pregnancy can worsen myotonia symptoms in up to 62% of cases but this resolves in 98% after pregnancy although it can sometimes take several months [75]. Symptoms may be more apparent in sodium channel compared with chloride channel–related myotonia [23]. Pharmacological treatments are usually discontinued during pregnancy either because of evidence of teratogenicity (e.g. carbamazepine [76], or acetazolamide [77]) or lack of evidence for safety. This contributes to the deterioration in symptoms. In severe cases, this needs to be balanced with the increase in risk to the mother and her offspring secondary to falls and reduced mobility. In particular, in the later stages of pregnancy, the risk/benefit ratio may be more in favour of treatment aiming to find the lowest effective dose [78]. Options include lamotrigine and mexiletine. A large amount of data in relation to lamotrigine in pregnancy is available from experience in epilepsy [74, 76] suggesting an increase in the risk of autism but no association with major congenital malformation. Mexiletine crosses the placenta and is excreted in breast milk but animal studies have not shown signs of teratogenicity. Cases have been reported of using mexiletine during pregnancy without adverse event [79, 80].

During labour as well as generally for any surgical intervention, it is beneficial that the room temperature is not cold and care must be taken to maintain normothermia (e.g. by using forced air warming devices and warmed IV fluids if necessary) and normal hydration. Where possible, it is advisable that the mother be able to mobilise and walk around if she wishes during labour as prolonged bed rest can exacerbate myotonia. Monitoring of electrolytes in particular serum potassium levels to prevent hyperkalaemia may be necessary. The depolarising muscle relaxant succinylcholine can elicit a myotonic crisis including masseter and laryngospasms potentially compromising airways and is therefore contraindicated. Use of non-depolarising muscle relaxants should be minimised in particular in those patients with persistent or intermittent attacks of weakness (PMC and HyperPP). If required, short-acting agents and lower doses are recommended as anticholinesterase drugs to reverse neuromuscular blockage can precipitate myotonia. Volatile anaesthetics as well as propofol are safe to use; indeed, the latter may have some direct antimyotonic properties [81].

## Treatment of periodic paralysis

### Pathophysiology

In all types of periodic paralysis, attacks of muscle weakness are characterised by partial depolarisation of the muscle fibre membrane causing the majority of voltage-gated sodium channels, Na_v_1.4, to become inactivated therefore rendering the membrane inexcitable (for review, see [82•]). In hyperkalaemic periodic paralysis, this is due to gain of function mutations in the voltage-gated sodium channel genes themselves, causing an increased inward sodium current leading to depolarization. The defect in ATS involves the inward rectifying potassium channel Kir2.1. This class of channels carries the majority of the potassium current at hyperpolarised membrane potential and loss of function mutations destabilise the resting membrane potential and allow for partial depolarization. The final and most common mechanism is the gating pore leak—a small inward cation current created due to substitution of positively charged amino acids within the voltage-sensing segments of the calcium and sodium channels. This current does not change the resting membrane potential dramatically but alters the behavior in response to low extracellular potassium. Under normal circumstances, the muscle membrane can tolerate quite low levels of potassium before exceeding the capacity of inward rectifying potassium channels to counteract the rising leak current introduced by the increasing hyperpolarisation of the membrane. Eventually, however, with extremely low serum potassium concentration, the membrane undergoes paradoxical partial depolarization, a state where voltage-gated sodium channels are inactivated and the membrane is inexcitable. In patients with HypoPP mutations, this state can be triggered with relatively mild lowering of serum potassium.

Treatment of periodic paralysis involves both interventions to abort or ameliorate an acute attack of weakness as well as preventative strategies.

#### Diet and lifestyle

The strongest trigger of a paralytic attack in any form of periodic paralysis is excessive exercise followed by rest. The most important advice is therefore for patients to adhere to ‘warming-up’ as well as ‘warming-down’ with any type of exercise. If possible, activities should be scheduled earlier in the day and avoided before bedtime. Gentle exercise however, e.g. walking, can be utilised to abort an impending attack if the onset is recognised at an early stage. Other common triggers are alcohol, stress and lack of sleep, perhaps part of the reason why attacks tend to be more frequent during adolescent and young adulthood [10•, 83].

Diet is another modifiable trigger for many. Patients with HypoPP should avoid high carbohydrate loads, which stimulate insulin secretion, which in turn can drive serum potassium from the extra to the intracellular compartment and trigger muscle weakness. In contrast, patients with HyperPP should avoid potassium-rich diets and fasting. Carbohydrate-containing snacks can be beneficial in acute HyperPP attacks but are not recommended as a long-term treatment strategy. Diet advice for patients with ATS generally follows HypoPP advice. A food and lifestyle diary can be useful in patients with frequent attacks to identify triggers.

#### Pharmacologic treatment

1. Carbonic anhydrase inhibitor
  Acetazolamide and dichlorphenamide are carbonic anhydrase inhibitors (CAI) and are widely used for prevention of periodic paralysis attacks. The enzyme carbonic anhydrase converts carbonic acid to carbon dioxide and water in the renal tubular lumen and carbon dioxide and water to carbonic acid in the proximal convoluted tubule cell. CAI decrease carbon dioxide reabsorption, as well as proton formation in proximal convoluted tubule (PCT), and increase urinary bicarbonate excretion leading to a metabolic acidosis.
  Acetazolamide and later dichlorphenamide have been routinely used for HypoPP and HyperPP for many years [10•]. The mechanism of action has been debated. The original rationale for using the drug was its effect as a mild potassium-wasting diuretic which was thought to be beneficial in hyperkalaemic periodic paralysis [84] but it was later found to be beneficial in all types of familial periodic paralysis [85] suggesting a different mechanism with metabolic acidosis and/or a direct effect on calcium-activated potassium channels suspected [10•, 86]. Alteration of intra- and extracellular pH can affect channel gating and this effect can be influenced in addition by mutations (e.g. lower intracellular pH resulted in normalising pH-sensitive Na^+^ channel behavior in the R669H and R672H HypoPP mutations [87]). Indirect evidence for the mechanism of action of CAI comes from a recent study which showed that prolonged and excessive exercise may produce local acidosis and abrupt recovery can induce a transient loss of force in mouse models [88•].
  Although acetazolamide was the first CAI used successfully in periodic paralysis and despite (or perhaps because) it remains easily accessible and affordable because it is licenced for other indications, the level of evidence is better for dichlorphenamide with two randomised controlled trials showing efficacy for attack reduction [89, 90] while evidence for acetazolamide is mostly based on case reports and series and non-randomised single-blind studies [10•, 91]. Both drugs work well for many patients although a higher response rate was seen in HypoPP patients with *CACNA1S* mutations compared with those with *SCN4A* mutations (56% versus 15% response, respectively), likely related to specific genotype (type of amino acid substitution) rather than the gene involved [92]. The most common side effect apart from transitory paraesthesiae is slowed cognition which led to dose reduction and some drop outs in the randomised controlled trials of dichlorphenamide. A worsening of attacks has been reported in particular associated with HypoPP sodium channel mutations [89, 93]. A small placebo-controlled cross-over trial of eight patients with HypoPP and acetazolamide demonstrated overall improved muscle strength with treatment but was not powered or of sufficient length to assess attack frequency [94].
  Some of the CAI’s side effects such as paresthesia, fatigue and mild cognitive disturbance can be minimised by gradual titration of the dose and using the minimum effective dose if necessary by introducing combination therapy with diuretics [95]. However, CAI use carries a clear risk of nephrolithiasis, with acetazolamide reported to show an increase by 15% within 1.5 years after initial treatment, which was not related to dosage [96]. A baseline renal ultrasound scan should therefore be performed before commencing CAI therapy and annual monitoring may reduce acute presentation with ureteric obstruction from urolithiasis [45•].
2. Diuretics and potassium
  Diuretics that alter serum potassium levels have been used to treat periodic paralysis effectively. In HypoPP, a potassium-sparing diuretic such as spironolactone lowers the chance of hypokalaemia, and, as a result, can reduce HypoPP attacks [10•]. Many patients are also given regular potassium supplementation for the treatment of HypoPP although there are no controlled studies to support this practice.
  Conversely, HyperPP potassium-wasting diuretics, such as hydrochlorothiazide or bendroflumethiazide, prevent hyperkalaemia and are effective in reducing the number of HyperPP attacks [97].
  The use of diuretics and potassium supplement as prophylactic agents does require regular monitoring of serum electrolytes especially when initiating treatment and after dose alterations.
  More recently, bumetanide, a loop-diuretic agent and Na^+^/K^+^/Cl^−^ cotransporter (NKCC1) antagonist, has been identified as a potential new treatment for HypoPP. Mouse models of HypoPP (both Na_V_1.4 and Ca_V_1.1 mutants) demonstrated that bumetanide can inhibit Na-K-Cl cotransporter thereby limiting the intracellular chloride concentration rise associated with repeated action potentials, leading to muscle fibre membrane stabilisation in a low extracellular potassium state. This helped to prevent the development of weakness and could induce recovery of force during an established attack [98, 99].
  We have conducted a randomised, double-blind, placebo-controlled phase II clinical trial of bumetanide to rescue an attack of weakness in HypoPP (EudraCT number 2013-004195-36). This study included ten patients with symptomatic disease and genetically confirmed *CACNA1S* mutations. A localised attack of weakness was induced by 5 min of isometric abductor digit minimi exercise followed by rest and defined as 40% CMAP (compound muscle action potential) amplitude decrement compared with the peak CMAP amplitude during or after the exercise. The results showed no statistically significant difference in the mean CMAP percentage of peak amplitudes between the bumetanide group and the placebo group 1 h after drug intake (the main outcome measure) but two patients recovered from their attack of weakness with bumetanide compared with none in the placebo group. There were no severe adverse events following 2 mg bumetanide intake. The negative results may have been contributed to by small sample size, prolonged limb immobilisation, rescue rather than preventive strategy and relatively low dose compared with the animal experiments. Further future research is still warranted to study the possibility of this drug for the treatment of HypoPP.

#### Pregnancy and anaesthetic considerations

Periodic paralysis attacks can worsen during pregnancy further exacerbated by withdrawal of treatment due to safety concerns. The use of acetazolamide in pregnancy is controversial. Animal studies suggested teratogenic effects [100, 101] but data from a large series of 101 women treated for intracranial hypertension covering 158 pregnancies showed no convincing evidence for adverse effects even when used before the 13th week of gestation (documented in 50 pregnancies) [102]. Less is known about dichlorphenamide but in general, metabolic acidosis can potentially affect fetal bone growth and fetal development. For labour and surgical intervention, similar advice as for myotonia exists with minimising length of inactivity, maintaining normothermia and meticulous attention to potassium levels all important factors [103]. Perioperative management of stress and anxiety may reduce the risk of attacks. Succinylcholine is contraindicated in HyperPP as it can trigger myotonic symptoms and hyperkalaemia. Short-acting non-depolarising neuromuscular blocking agents are preferable for all types of periodic paralysis and in HyperPP in particular anticholinesterases are avoided because of the risk of exacerbation of myotonia. Intravenous dextrose in HyperPP is useful to cover the fasting period while large carbohydrate loading should be avoided in HypoPP. Based on a couple of case reports of malignant hyperthermia reactions in patients with HypoPP, it is advisable to use malignant hyperthermia trigger-free anaesthetics [104]. ECG monitoring is essential for all periodic paralysis patients but in particular for ATS.

#### Emergency therapy

Among the muscle channelopathies, HypoPP and ATS patients are most likely to present to the emergency department with hypokalaemia-related paralysis. To abort an acute attack in HypoPP and ATS associated with hypokalaemia, oral K^+^ at a dose of 0.5–1 mEq/kg up to 200 mEq/24 h can be given. In the majority of cases, oral and non-slow-release formulations are preferrable [10•]. Intravenous potassium up to maximum of 20 mEq/h using a non-glucose or saline containing solution (e.g. mannitol) should be restricted to cases with severe arrhythmias or respiratory compromise and requires potassium and ECG monitoring. It is important to remember that hypokalaemia during a HypoPP attack is due to a shift of the potassium from the extra- to the intracellular compartment. Patients therefore are not potassium deficient and inducing hyperkalaemia is a particular risk with aggressive replacement therapy especially if there is inadequate monitoring of serum potassium levels and ECG changes.

Inhaled beta-agonists such as salbutamol have been used to treat HyperPP attacks as well as standard intravenous potassium lowering treatment involving glucose and insulin [105, 106].

## Conclusion

Treatment options for skeletal muscle channelopathies have expanded over time facilitated by our increased understanding of the underlying pathophysiology. The lack of Level I evidence for many agents is perhaps not surprising given the rarity of these conditions but newer approaches to trial designs are promising. Patients are best looked after by specialists in neuromuscular conditions but a good relationship with primary care physicians is essential to achieve optimal care and monitoring of their condition and their treatment. Management during pregnancy continues to be suboptimal due to lack of evidence for safety for most drugs. A number of clinical questions remain unanswered such as whether periodic paralysis attack prevention reduces the incidence or severity of permanent muscle weakness later in life, and the direct comparison between drugs (i.e. acetazolamide versus dichlorphenamide) and whether combination therapy is better than the use of single agents.

## References

[1] Horga A, Raja Rayan DL, Matthews E, Sud R, Fialho D, Durran SCM (2013). Prevalence study of genetically defined skeletal muscle channelopathies in England. Neurology..

[2] Stunnenberg BC, Raaphorst J, Deenen JCW, Links TP, Wilde AA, Verbove DJ (2018). Prevalence and mutation spectrum of skeletal muscle channelopathies in the Netherlands. Neuromuscul Disord.

[3] Stunnenberg BC, LoRusso S, Arnold WD, Barohn RJ, Cannon SC, Fontaine B, et al. Guidelines on clinical presentation and management of nondystrophic myotonias. Muscle Nerve. 2020. 10.1002/mus.26887. 10.1002/mus.26887 PMC8117169 32270509

[4] Sie LTL, Stunnenberg B, Trip J, Ginjaar IB, Drost G (2009). G.P.14.10 Clinical experience with acetazolamide treatment in children; good response in both sodium and chloride channelopathies. Neuromuscul Disord.

[5] Singh RR, Tan SV, Hanna MG, Robb SA, Clarke A, Jungbluth H (2014). Mutations in SCN4A: a rare but treatable cause of recurrent life-threatening laryngospasm. Paediatrics..

[6] Portaro S, Rodolico C, Sinicropi S, Musumeci O, Valenzise M, Toscano A. Flecainide-responsive myotonia permanens with SNEL onset: a new case and literature review. Paediatrics. 2016;137. 10.1542/peds.2015-3289 26944947

[7] Hammarén E, Kjellby-Wendt G, Lindberg C (2005). Quantification of mobility impairment and self-assessment of stiffness in patients with myotonia congenita by the physiotherapist. Neuromuscul Disord.

[8] Bohannon RW (1995). Sit-to-stand test for measuring performance of lower extremity muscles. Percept Mot Skills.

[9] Podsiadlo D, Richardson S (1991). The timed “Up & Go”: a test of basic functional mobility for frail elderly persons. J Am Geriatr Soc.

[10] • Statland JM, Fontaine B, Hanna MG, Johnson NE, Kissel JT, Sansone VA, et al. Review of the diagnosis and treatment of periodic paralysis. Muscle Nerve. 2018;57:522–30. **Recent review of periodic paralysis**. 10.1002/mus.26009 PMC5867231 29125635

[11] McManis PG, Lambert EH, Daube JR (1986). The exercise test in periodic paralysis. Muscle Nerve.

[12] • Mazzanti A, Guz D, Trancuccio A, Pagan E, Kukavica D, Chargeishvili T, et al. Natural history and risk stratification in Andersen-Tawil syndrome type 1. J Am Coll Cardiol. 2020;75:1772–84. **Reveals higher than expected risk of life-threatening arrhythmias in ATS**. 10.1016/j.jacc.2020.02.033 32299589

[13] Matthews E, Silwal A, Sud R, Hanna MG, Manzur AY, Muntoni F (2017). Skeletal muscle channelopathies: rare disorders with common pediatric symptoms. J Pediatr.

[14] Zaharieva IT, Thor MG, Oates EC, van Karnebeek C, Hendson G, Blom E (2016). Loss-of-function mutations in SCN4A cause severe foetal hypokinesia or “classical” congenital myopathy. Brain..

[15] Tsujino A, Maertens C, Ohno K, Shen X-M, Fukuda T, Harper CM (2003). Myasthenic syndrome caused by mutation of the SCN4A sodium channel. Proc Natl Acad Sci USA.

[16] Arnold WD, Feldman DH, Ramirez S, He L, Kassar D, Quick A (2015). Defective fast inactivation recovery of Nav 1.4 in congenital myasthenic syndrome. Ann Neurol.

[17] •• Männikkö R, Wong L, Tester DJ, Thor MG, Sud R, Kullmann DM, et al. Dysfunction of NaV1.4, a skeletal muscle voltage-gated sodium channel, in sudden infant death syndrome: a case-control study. Lancet. 2018;391:1483–92. **Suggests a role of skeletal muscle sodium channel mutations in sudden infant death**. 10.1016/S0140-6736(18)30021-7 PMC5899997 29605429

[18] Morrow JM, Matthews E, Raja Rayan DL, Fischmann A, Sinclair CDJ, Reilly MM (2013). Muscle MRI reveals distinct abnormalities in genetically proven non-dystrophic myotonias. Neuromuscul Disord.

[19] Maggi L, Brugnoni R, Canioni E, Maccagnano E, Bernasconi P, Morandi L (2015). Imaging alterations in skeletal muscle channelopathies: a study in 15 patients. Acta Myol.

[20] Jeong H-N, Yi JS, Lee YH, Lee JH, Shin HY, Choi Y-C (2018). Lower-extremity magnetic resonance imaging in patients with hyperkalemic periodic paralysis carrying the SCN4A mutation T704M: 30-month follow-up of seven patients. Neuromuscul Disord.

[21] Andersen G, Løkken N, Vissing J (2017). Aerobic training in myotonia congenita: effect on myotonia and fitness. Muscle Nerve.

[22] Stefanetti RJ, Blain A, Jimenez-Moreno C, Errington L, Shiau Ng Y, McFarland R, et al. Measuring the effects of exercise in neuromuscular disorders: a systematic review and meta-analyses [Internet]. Rochester, NY: Social Science Research Network; 2019 Oct. Report No.: ID 3469742. Available from: https://papers.ssrn.com/abstract=3469742. Accessed 21 Jun 2020. 10.12688/wellcomeopenres.15825.1 PMC7331112 32671231

[23] Trivedi JR, Bundy B, Statland J, Salajegheh M, Rayan DR, Venance SL (2013). Non-dystrophic myotonia: prospective study of objective and patient reported outcomes. Brain..

[24] D’Mello S, Shum L (2016). A review of the use of mexiletine in patients with myotonic dystrophy and non-dystrophic myotonia. Eur J Hosp Pharm.

[25] Nakagawa H, Munakata T, Sunami A. Mexiletine block of voltage-gated sodium channels: isoform- and state-dependent drug–pore interactions. Mol Pharmacol. American Society for Pharmacology and Experimental Therapeutics. 2019;95:236–44. 10.1124/mol.118.114025 30593458

[26] Desaphy JF, De Luca A, Tortorella P, De Vito D, George AL, Conte CD (2001). Gating of myotonic Na channel mutants defines the response to mexiletine and a potent derivative. Neurology..

[27] Wang GK, Russell C, Wang S-Y (2004). Mexiletine block of wild-type and inactivation-deficient human skeletal muscle hNav1.4 Na + channels. J Physiol Lond.

[28] De Luca A, Pierno S, Liantonio A, Desaphy J-F, Natuzzi F, Didonna MP (2004). New potent mexiletine and tocainide analogues evaluated in vivo and in vitro as antimyotonic agents on the myotonic ADR mouse. Neuromuscul Disord.

[29] Mohammadi B, Jurkat-Rott K, Alekov A, Dengler R, Bufler J, Lehmann-Horn F (2005). Preferred mexiletine block of human sodium channels with IVS4 mutations and its pH-dependence. Pharmacogenet Genomics.

[30] Statland JM, Bundy BN, Wang Y, Rayan DR, Trivedi JR, Sansone VA (2012). Mexiletine for symptoms and signs of myotonia in nondystrophic myotonia: a randomized controlled trial. JAMA..

[31] •• Stunnenberg BC, Raaphorst J, Groenewoud HM, Statland JM, Griggs RC, Woertman W, et al. Effect of mexiletine on muscle stiffness in patients with nondystrophic myotonia evaluated using aggregated N-of-1 trials. JAMA. 2018;320:2344–53. **Elegant trial design confirming efficacy of mexiletine in NDM**. 10.1001/jama.2018.18020 PMC6583079 30535218

[32] Ginanneschi F, Mignarri A, Lucchiari S, Ulzi G, Comi GP, Rossi A (2017). Neuromuscular excitability changes produced by sustained voluntary contraction and response to mexiletine in myotonia congenita. Neurophysiol Clin.

[33] Kwieciński H, Ryniewicz B, Ostrzycki A (1992). Treatment of myotonia with antiarrhythmic drugs. Acta Neurol Scand.

[34] Suetterlin KJ, Bugiardini E, Kaski JP, Morrow JM, Matthews E, Hanna MG (2015). Long-term safety and efficacy of mexiletine for patients with skeletal muscle channelopathies. JAMA Neurol.

[35] Romman A, Salama-Hanna J, Dwivedi S (2018). Mexiletine usage in a chronic pain clinic: indications, tolerability, and side effects. Pain Physician.

[36] Nakatani Y, Masuko H, Amano T (2013). Effect of lamotrigine on Na(v)1.4 voltage-gated sodium channels. J Pharmacol Sci.

[37] Errington AC, Stöhr T, Heers C, Lees G (2008). The investigational anticonvulsant lacosamide selectively enhances slow inactivation of voltage-gated sodium channels. Mol Pharmacol.

[38] Skov M, de Paoli FV, Nielsen OB, Pedersen TH (2017). The anti-convulsants lacosamide, lamotrigine, and rufinamide reduce myotonia in isolated human and rat skeletal muscle. Muscle Nerve.

[39] • Andersen G, Hedermann G, Witting N, Duno M, Andersen H, Vissing J. The antimyotonic effect of lamotrigine in non-dystrophic myotonias: a double-blind randomized study. Brain. 2017;140:2295–305. **Demonstrating efficacy of lamotrigine for myotonia**. 10.1093/brain/awx192 29050397

[40] Griggs RC, Moxley RT, Riggs JE, Engel WK (1978). Effects of acetazolamide on myotonia. Ann Neurol.

[41] Markhorst JM, Stunnenberg BC, Ginjaar IB, Drost G, Erasmus CE, Sie LTL (2014). Clinical experience with long-term acetazolamide treatment in children with nondystrophic myotonias: a three-case report. Pediatr Neurol.

[42] Moreira SD, Barreto R, Roriz JM (2015). Becker myotonia—a recently identified mutation in iberian descendants with apparent acetazolamide-responsive phenotype. Muscle Nerve.

[43] Trudell RG, Kaiser KK, Griggs RC (1987). Acetazolamide-responsive myotonia congenita. Neurology..

[44] Eguchi H, Tsujino A, Kaibara M, Hayashi H, Shirabe S, Taniyama K (2006). Acetazolamide acts directly on the human skeletal muscle chloride channel. Muscle Nerve.

[45] • Suetterlin KJ, Vivekanandam V, James N, Sud R, Holmes S, Fialho D, et al. Annual renal ultrasound may prevent acute presentation with acetazolamide-associated urolithiasis. Neurol Clin Pract. 2019. 10.1212/CPJ.0000000000000761. **Audit data of acetazolamide use in skeletal muscle channelopathies**. 10.1212/CPJ.0000000000000761 PMC8101321 33968492

[46] Sheela SR (2000). Myotonia congenita: response to carbamazepine. Indian Pediatr.

[47] Lyons MJ, Duron R, Molinero I, Sangiuolo F, Holden KR (2010). Novel CLCN1 mutation in carbamazepine-responsive myotonia congenita. Pediatr Neurol.

[48] Berardinelli A, Gorni K, Orcesi S (2000). Response to carbamazepine of recessive-type myotonia congenita. Muscle Nerve.

[49] Savitha MR (2006). Krishnamurthy B, Hyderi A, Farhan-Ul-Haque null, Ramachandra NB. Myotonia congenita--a successful response to carbamazepine. Indian J Pediatr.

[50] Sechi GP, Traccis S, Durelli L, Monaco F, Mutani R (1983). Carbamazepine versus diphenylhydantoin in the treatment of myotonia. Eur Neurol.

[51] Aoike F, Takahashi MP, Sakoda S (2006). Class Ic antiarrhythmics block human skeletal muscle Na channel during myotonia-like stimulation. Eur J Pharmacol.

[52] Desaphy J-F, Carbonara R, Costanza T, Conte CD (2014). Preclinical evaluation of marketed sodium channel blockers in a rat model of myotonia discloses promising antimyotonic drugs. Exp Neurol.

[53] Echt DS, Liebson PR, Mitchell LB, Peters RW, Obias-Manno D, Barker AH (1991). Mortality and morbidity in patients receiving encainide, flecainide, or placebo. The Cardiac Arrhythmia Suppression Trial. N Engl J Med.

[54] Terracciano C, Farina O, Esposito T, Lombardi L, Napolitano F, Blasiis PD (2018). Successful long-term therapy with flecainide in a family with paramyotonia congenita. J Neurol Neurosurg Psychiatry.

[55] Rosenfeld J, Sloan-Brown K, George AL (1997). A novel muscle sodium channel mutation causes painful congenital myotonia. Ann Neurol.

[56] Desaphy J-F, Modoni A, Lomonaco M, Camerino DC (2013). Dramatic improvement of myotonia permanens with flecainide: a two-case report of a possible bench-to-bedside pharmacogenetics strategy. Eur J Clin Pharmacol.

[57] • Farinato A, Altamura C, Imbrici P, Maggi L, Bernasconi P, Mantegazza R, et al. Pharmacogenetics of myotonic hNav1.4 sodium channel variants situated near the fast inactivation gate. Pharmacol Res. 2019;141:224–35. **In vitro study examining effect of*****SCN4A*****mutations on efficacy of mexiletine versus flecainide**. 10.1016/j.phrs.2019.01.004 30611854

[58] Desaphy J-F, Carbonara R, D’Amico A, Modoni A, Roussel J, Imbrici P (2016). Translational approach to address therapy in myotonia permanens due to a new SCN4A mutation. Neurology..

[59] Lehmann-Horn F, D’Amico A, Bertini E, Lomonaco M, Merlini L, Nelson KR (2017). Myotonia permanens with Nav1.4-G1306E displays varied phenotypes during course of life. Acta Myol.

[60] Cavalli M, Fossati B, Vitale R, Brigonzi E, Ricigliano VAG, Saraceno L (2018). Flecainide-induced Brugada syndrome in a patient with skeletal muscle sodium channelopathy: a case report with critical therapeutical implications and review of the literature. Front Neurol.

[61] Hawash AA, Voss AA, Rich MM (2017). Inhibiting persistent inward sodium currents prevents myotonia. Ann Neurol.

[62] Novak KR, Norman J, Mitchell JR, Pinter MJ, Rich MM (2015). Sodium channel slow inactivation as a therapeutic target for myotonia congenita. Ann Neurol.

[63] El-Bizri N, Kahlig KM, Shyrock JC, George AL, Belardinelli L, Rajamani S (2011). Ranolazine block of human Na v 1.4 sodium channels and paramyotonia congenita mutants. Channels (Austin).

[64] Kahlig KM, Hirakawa R, Liu L, George AL, Belardinelli L, Rajamani S (2014). Ranolazine reduces neuronal excitability by interacting with inactivated states of brain sodium channels. Mol Pharmacol.

[65] Lossin C (2013). Nav 1.4 slow-inactivation: is it a player in the warm-up phenomenon of myotonic disorders?. Muscle Nerve.

[66] • Arnold WD, Kline D, Sanderson A, Hawash AA, Bartlett A, Novak KR, et al. Open-label trial of ranolazine for the treatment of myotonia congenita. Neurology. 2017;89:710–3. **Ranolazine trial for myotonia congenita**. 10.1212/WNL.0000000000004229 PMC5562961 28710329

[67] • Lorusso S, Kline D, Bartlett A, Freimer M, Agriesti J, Hawash AA, et al. Open-label trial of ranolazine for the treatment of paramyotonia congenita. Muscle Nerve. 2019;59:240–3. **Ranolazine trial for paramyotonia congenita**. 10.1002/mus.26372 PMC6340713 30390395

[68] Heatwole CR, Statland JM, Logigian EL (2013). The diagnosis and treatment of myotonic disorders. Muscle Nerve.

[69] • Desaphy J-F, Farinato A, Altamura C, De Bellis M, Imbrici P, Tarantino N, et al. Safinamide’s potential in treating nondystrophic myotonias: inhibition of skeletal muscle voltage-gated sodium channels and skeletal muscle hyperexcitability in vitro and in vivo. Exp Neurol. 2020;328:113287. **Potential new antimyotnic agent investigated**. 10.1016/j.expneurol.2020.113287 32205118

[70] Lamanauskas N, Nistri A (2008). Riluzole blocks persistent Na+ and Ca2+ currents and modulates release of glutamate via presynaptic NMDA receptors on neonatal rat hypoglossal motoneurons in vitro. Eur J Neurosci.

[71] Skov M, De Paoli FV, Lausten J, Nielsen OB, Pedersen TH (2015). Extracellular magnesium and calcium reduce myotonia in isolated ClC-1 chloride channel-inhibited human muscle. Muscle Nerve.

[72] Skov M, Riisager A, Fraser JA, Nielsen OB, Pedersen TH (2013). Extracellular magnesium and calcium reduce myotonia in ClC-1 inhibited rat muscle. Neuromuscul Disord.

[73] Mankodi A, Grunseich C, Skov M, Cook L, Aue G, Purev E (2015). Divalent cation-responsive myotonia and muscle paralysis in skeletal muscle sodium channelopathy. Neuromuscul Disord.

[74] Veroniki AA, Cogo E, Rios P, Straus SE, Finkelstein Y, Kealey R (2017). Comparative safety of anti-epileptic drugs during pregnancy: a systematic review and network meta-analysis of congenital malformations and prenatal outcomes. BMC Med.

[75] Snyder Y, Donlin-Smith C, Snyder E, Pressman E, Ciafaloni E (2015). The course and outcome of pregnancy in women with nondystrophic myotonias. Muscle Nerve.

[76] Weston J, Bromley R, Jackson CF, Adab N, Clayton-Smith J, Greenhalgh J (2016). Monotherapy treatment of epilepsy in pregnancy: congenital malformation outcomes in the child. Cochrane Database Syst Rev.

[77] Al-Saleem AI, Al-Jobair AM (2016). Possible association between acetazolamide administration during pregnancy and multiple congenital malformations. Drug Des Devel Ther.

[78] Rudnik-Schöneborn S, Witsch-Baumgartner M, Zerres K (2016). Influences of pregnancy on different genetic subtypes of non-dystrophic myotonia and periodic paralysis. Gynecol Obstet Investig.

[79] Yano M, Nishida Y, Kai K, Ishii T, Takahashi N, Narahara H (2017). Long QT syndrome in pregnancy: a successful case of ICD implantation during the prenatal period. J Obstet Gynaecol.

[80] Cordina R, McGuire MA (2010). Maternal cardiac arrhythmias during pregnancy and lactation. Obstet Med.

[81] Bandschapp O, Ginz HF, Soule CL, Girard T, Urwyler A, Iaizzo PA (2009). In vitro effects of propofol and volatile agents on pharmacologically induced chloride channel myotonia. Anesthesiology..

[82] • Cannon SC. Sodium channelopathies of skeletal muscle. Handb Exp Pharmacol. 2018;246:309–30. **Detailed review of pathophysiology of skeletal muscle sodium channelopathies.** 10.1007/164_2017_52 PMC5866235 28939973

[83] Venance SL, Cannon SC, Fialho D, Fontaine B, Hanna MG, Ptacek LJ, et al. The primary periodic paralyses: diagnosis, pathogenesis and treatment. Brain Oxford Academic. 2006;129:8–17. 10.1093/brain/awh639 16195244

[84] McArdle B. Metabolic myopathies: the glycogenoses affecting muscle, and hypo- and hyperkalemic periodic paralysis. Am J Med. 1963;35:661–72. 10.1016/0002-9343(63)90137-2 14076019

[85] Resnick JS, Engel WK, Griggs RC, Stam AC (1968). Acetazolamide prophylaxis in hypokalemic periodic paralysis. N Engl J Med.

[86] Tricarico D, Mele A, Conte CD (2006). Carbonic anhydrase inhibitors ameliorate the symptoms of hypokalaemic periodic paralysis in rats by opening the muscular Ca2+-activated-K+ channels. Neuromuscul Disord.

[87] Kuzmenkin A, Muncan V, Jurkat-Rott K, Hang C, Lerche H, Lehmann-Horn F (2002). Enhanced inactivation and pH sensitivity of Na+ channel mutations causing hypokalaemic periodic paralysis type II. Brain..

[88] • Mi W, Wu F, Quinonez M, DiFranco M, Cannon SC. Recovery from acidosis is a robust trigger for loss of force in murine hypokalemic periodic paralysis. J Gen Physiol. 2019;151:555–66. **The Rockefeller University Press. Provides evidence to explain the delayed onset of weakness after exercise in PP**. 10.1085/jgp.201812231 PMC6445579 30733232

[89] Sansone VA, Burge J, McDermott MP, Smith PC, Herr B, Tawil R (2016). Randomized, placebo-controlled trials of dichlorphenamide in periodic paralysis. Neurology..

[90] Tawil R, McDermott MP, Brown R, Shapiro BC, Ptacek LJ, McManis PG (2000). Randomized trials of dichlorphenamide in the periodic paralyses. Ann Neurol.

[91] Sansone V, Meola G, Links T, Panzeri M, Rose MR. Treatment for periodic paralysis. Cochrane Database of Systematic Reviews [Internet]. John Wiley & Sons, Ltd.; 2008 [cited 2020 Mar 10]; Available from: https://www.cochranelibrary.com/cdsr/doi/10.1002/14651858.CD005045.pub2/full. Accessed 21 Jun 2020. 10.1002/14651858.CD005045.pub2 18254068

[92] Matthews E, Portaro S, Ke Q, Sud R, Haworth A, Davis MB (2011). Acetazolamide efficacy in hypokalemic periodic paralysis and the predictive role of genotype. Neurology..

[93] Ikeda K, Iwasaki Y, Kinoshita M, Yabuki D, Igarashi O, Ichikawa Y (2002). Acetazolamide-induced muscle weakness in hypokalemic periodic paralysis. Intern Med.

[94] Links TP, Zwarts MJ, Oosterhuis HJ. Improvement of muscle strength in familial hypokalaemic periodic paralysis with acetazolamide. J Neurol Neurosurg Psychiatry. BMJ Publishing Group Ltd. 1988;51:1142–5. 10.1136/jnnp.51.9.1142 PMC1033016 3066848

[95] Lichter PR (1981). Reducing side effects of carbonic anhydrase inhibitors. Ophthalmology..

[96] Au JN, Waslo CS, McGwin G, Huisingh C, Tanne E (2016). Acetazolamide-induced nephrolithiasis in idiopathic intracranial hypertension patients. J Neuroophthalmol.

[97] Akaba Y, Takahashi S, Sasaki Y, Kajino H (2018). Successful treatment of normokalemic periodic paralysis with hydrochlorothiazide. Brain Dev.

[98] Wu F, Mi W, Cannon SC (2013). Beneficial effects of bumetanide in a CaV1.1-R528H mouse model of hypokalaemic periodic paralysis. Brain..

[99] Wu F, Mi W, Cannon SC (2013). Bumetanide prevents transient decreases in muscle force in murine hypokalemic periodic paralysis. Neurology..

[100] Kojima N, Naya M, Makita T. Effects of maternal acetazolamide treatment on body weights and incisor development of the fetal rat. J Vet Med Sci. 1999;61:143–7. 10.1292/jvms.61.143 10081752

[101] Holmes LB, Kawanishi H, Munoz A. Acetazolamide: maternal toxicity, pattern of malformations, and litter effect. Teratology. 1988;37:335–42. 10.1002/tera.1420370407 3394109

[102] Falardeau J, Lobb BM, Golden S, Maxfield SD, Tanne E (2013). The use of acetazolamide during pregnancy in intracranial hypertension patients. J Neuroophthalmol.

[103] Bandschapp O, Iaizzo PA (2013). Pathophysiologic and anesthetic considerations for patients with myotonia congenita or periodic paralyses. Paediatr Anaesth.

[104] Marchant CL, Ellis FR, Halsall PJ, Hopkins PM, Robinson RL (2004). Mutation analysis of two patients with hypokalemic periodic paralysis and suspected malignant hyperthermia. Muscle Nerve.

[105] Bendheim PE, Reale EO, Berg BO (1985). beta-Adrenergic treatment of hyperkalemic periodic paralysis. Neurology..

[106] Hanna MG, Stewart J, Schapira AH, Wood NW, Morgan-Hughes JA, Murray NM (1998). Salbutamol treatment in a patient with hyperkalaemic periodic paralysis due to a mutation in the skeletal muscle sodium channel gene (SCN4A). J Neurol Neurosurg Psychiatry.

